# Coexisting spin and Rabi oscillations at intermediate time regimes in electron transport through a photon cavity

**DOI:** 10.3762/bjnano.10.61

**Published:** 2019-03-01

**Authors:** Vidar Gudmundsson, Hallmann Gestsson, Nzar Rauf Abdullah, Chi-Shung Tang, Andrei Manolescu, Valeriu Moldoveanu

**Affiliations:** 1 Science Institute, University of Iceland, Dunhaga 3, IS-107 Reykjavik, Iceland; 2 Physics Department, College of Science, University of Sulaimani, Kurdistan Region, Iraq; 3 Komar Research Center, Komar University of Science and Technology, Sulaimani, Kurdistan Region, Iraq; 4 Department of Mechanical Engineering, National United University, Miaoli 36003, Taiwan; 5 School of Science and Engineering, Reykjavik University, Menntavegur 1, IS-101 Reykjavik, Iceland; 6 National Institute of Materials Physics, PO Box MG-7, Bucharest-Magurele, Romania

**Keywords:** electron transport, interactions, photon cavity, photon-dressed electron states, time dependent

## Abstract

In this work, we theoretically model the time-dependent transport through an asymmetric double quantum dot etched in a two-dimensional wire embedded in a far-infrared (FIR) photon cavity. For the transient and the intermediate time regimes, the current and the average photon number are calculated by solving a Markovian master equation in the dressed-states picture, with the Coulomb interaction also taken into account. We predict that in the presence of a transverse magnetic field the interdot Rabi oscillations appearing in the intermediate and transient regime coexist with slower non-equilibrium fluctuations in the occupation of states for opposite spin orientation. The interdot Rabi oscillation induces charge oscillations across the system and a phase difference between the transient source and drain currents. We point out a difference between the steady-state correlation functions in the Coulomb blocking and the photon-assisted transport regimes.

## Introduction

Experimental [1–6] and theoretical [7–11] interest is growing in electron transport through semiconductor systems in photon cavities. The success of circuit quantum electrodynamics (QED) devices with superconducting quantum bits coupled to microwave cavities has pushed for the evolution of hybrid mesoscopic circuits combining nanoconductors and metallic reservoirs [12]. Eventually, this effort might lead to the evolution of devices active in the challenging terahertz regime, which would open up novel possibilities [12]. This has led us to consider particular aspects of the electron–photon interaction on quantum transport in the far-infrared (FIR) regime.

The time-dependent electronic transport through a two-dimensional (2D) nanosystem patterned in a GaAs heterostructure, which is in turn embedded in a three-dimensional (3D) FIR photon cavity, generally displays three regimes: i) The switching transient regime in which electrons tunnel through the system but their interactions with the photons have not had time to affect the transport yet; ii) the intermediate regime during which the electron–photon coupling plays an important role in bringing the system to a steady state; and iii) the stationary regime with coexisting radiative transitions and photon-assisted tunneling [9]. The characteristic time of the transient and the intermediate regime depends on the the ratio of the system lead coupling and the electron–photon coupling in addition to the shape or geometry of the central system and the photon energy [13]. The formation of very slow Rabi-like spin-flip transitions promoted by the interplay of tunneling and spin orbit interactions in a system of double quantum dots has been studied by Khomitsky et al. [14].

In earlier publications we have shown how a Rabi oscillation can be detected in the transport of electrons through an electronic system in a 3D photon cavity, in the transient regime directly from the charge current [15], and in the steady state through the Fourier power spectrum of the current current correlation function [16]. Here, we will analyze the intermediate time regime and show that oscillations of the transport current in time still reveal Rabi oscillations, but in a complex many-level system other oscillations can be present. In particular we find that for a weak Rabi splitting the even weaker Zeeman spin splitting caused by a small external magnetic field plays a role in the transport, but only in this regime dominated by strong nonequilibrium processes.

In the earlier calculations the central system was a short quantum wire with parallel quantum dots of the same shape. The anisotropy of the system makes the first excitation of the even parity one-electron ground state to be an odd parity state with respect to the axis of the quantum dots, the *y*-axis. Subsequently, *y*-polarized cavity photons couple the two states strongly through the paramagnetic electron–photon interaction, but only weakly through the diamagnetic interaction. On the other hand, *x*-polarized photons can only couple the two states weakly through the diamagnetic interaction. We thus observed two different Rabi oscillations depending on the polarization of the cavity field [9,15–17]. Here, we select an asymmetric system with slightly dissimilar quantum dots located at opposite ends of the short quantum wire. Consequently, the energy levels of each dot are different (or misaligned). The dots are well separated, such that the charge probability density distribution of the lowest one-electron energy states of each dot is almost entirely located in each dot. The lower one of them is the one-electron ground state of the system, and the other one is the first excited one-electron state state. We select the photon energy to establish a Rabi resonance between these two states. It is bound to be weak as it relies on the small charge overlap of the states, but it is also interesting as it promotes a charge oscillation over the entire length of the short quantum wire.

## System and Model

**Figure 1:**
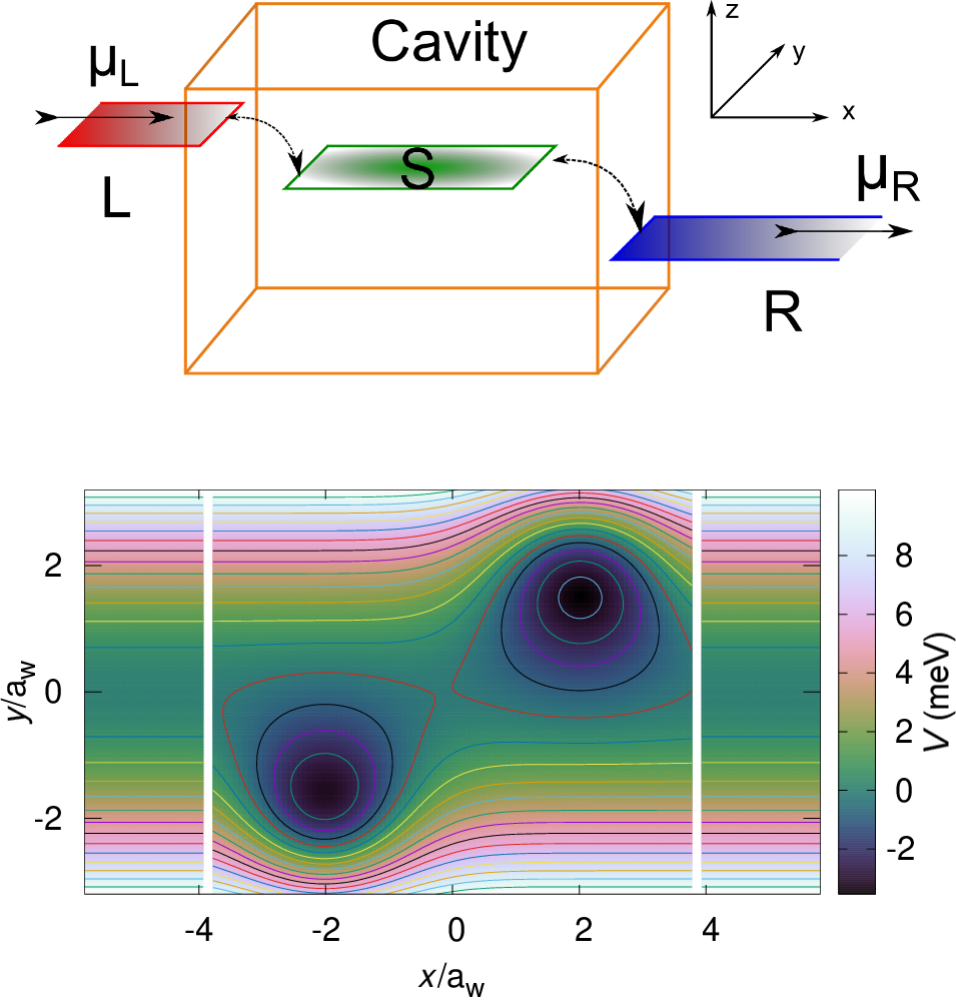
(Upper) Schematic for a short quantum wire in a 3D photon cavity coupled to semi-infinite external left (L) and right (R) leads with quasi 1D electron systems with chemical potentials μ_L_ and μ_R_, respectively. The electrons in the short quantum wire and the photons of the cavity comprise the central system (S). (Lower) The potential energy landscape defining the two asymmetrical quantum dots embedded in a short quantum wire of length *L_x_* = 180 nm ≈ 7.6*a_w_*, where *a_w_* = 23.8 nm is the effective magnetic length for transverse magnetic field *B*_ext_ = 0.1 T and parabolic confinement energy 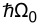 = 2.0 meV of the short wire and leads in the *y*-direction. The white gaps at *x* ≈ ±3.8*a_w_* indicate the onset of the semi-infinite leads. The right dot is slightly deeper and broader than the left dot.

We consider a short quantum wire of length *L* = 180 nm with two asymmetrically placed shallow quantum dots as is displayed in Figure 1.

The potential landscape defining the short quantum wire and dots is described by

[1]
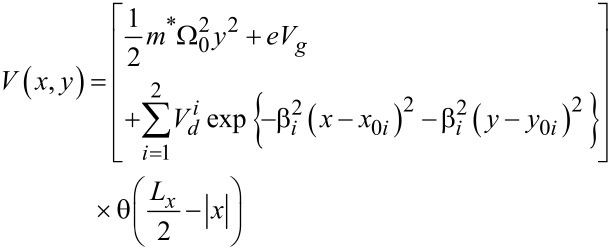


with 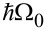 = 2.0 meV, 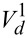 = −6.6 meV, 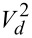 = −6.8 meV, β_1_ = 0.030 nm^−1^, β_2_ = 0.028 nm^−1^, *x*_01_ = −48 nm, *x*_02_ = +48 nm, *y*_01_ = −50 nm, *y*_02_ = +50 nm, *L_x_* = 180 nm, and θ the Heaviside unit step function. The plunger gate voltage *V_g_* shifts the states of the central system up or down in energy with respect to the bias window defined by the chemical potentials of the external leads to be described below.

The Hamiltonian of the closed central system, the electrons and the photons, in terms of field operators is

[2] $$\begin{array}{c} H_{\text{S}}=\int {\text{d}}^{2}r\psi^{†}\left(r\right)\left\{\frac{\pi^{2}}{2m^{*}}+V\left(r\right)\right\}\psi \left(r\right)+H_{\text{EM}}+H_{\text{Coul}}\\ +\frac{1}{c}\int {\text{d}}^{2}rj\left(r\right)\cdot A_{\gamma }+\frac{e}{2m^{*}c^{2}}\int {\text{d}}^{2}r\rho \left(r\right)A_{\gamma }^{2}, \end{array}$$

with

[3]
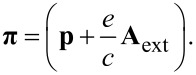


The static electron–electron Coulomb interaction is described by *H*_Coul_ with the kernel

[4]
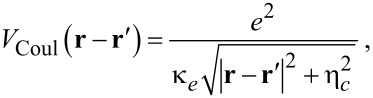


and a small regularization parameter η*_c_*/*a_w_* = 3 × 10^−7^ (*a_w_* is defined below). In the second line of the Hamiltonian (Equation 2) are the para- and the diamagnetic electron–photon interactions, respectively. **A**_ext_ (Equation 3) is a classical vector potential leading to a homogeneous, external, small magnetic field, *B*_ext_ = 0.1 T, directed along the *z*-axis, perpendicular to the two-dimensional quantum wire, inserted to break the spin and possible orbital degeneracies of the states in order to guarantee stability of the results. We use GaAs parameters with m^*^ = 0.067*m_e_*, κ*_e_* = 12.4, and g^*^ = −0.44. The small external magnetic field, *B*_ext_, and the parabolic confinement energy of the leads and the central system 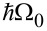 = 2.0 meV, together with the cyclotron frequency ω*_c_* = (*eB*_ext_)/(m^*^c) produce an effective characteristic confinement energy 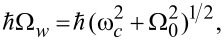 and an effective magnetic length 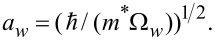 This characteristic length scale is approximately 23.8 nm for the parameters selected here. The Hamiltonian describing the single cavity photon mode is 
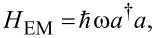
 with energy 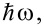 in terms of the cavity photon annihilation and creation operators, *a*^†^ and *a*. We model a rectangular 3D photon cavity (*x*,*y*,*z*) 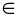 {[−*a*_c_/2,*a*_c_/2] × [−*a*_c_/2,*a*_c_/2] × [−*d*_c_/2,*d*_c_/2]} with the short quantum wire located in the center of the *z* = 0 plane.

For the Coulomb gauge used here the polarization of the electric field of the cavity photons parallel to the transport in the *x*-direction (with the unit vector **e***_x_*) is realized in the TE_011_ mode, or perpendicular to the transport (defined by the unit vector **e***_y_*) in the TE_101_ mode. The two modes of the quantized vector potential for the cavity field can be expressed as (in a stacked notation)

[5]
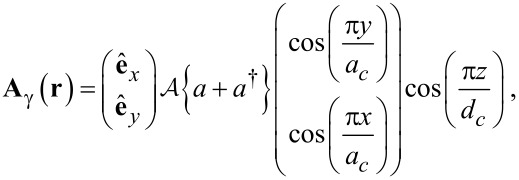


with the strength of the vector potential, 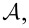 and the electron–photon coupling constant related by 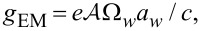 leading to a dimensionless polarization tensor

[6]
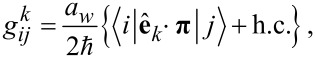


to be evaluated, where *k* = *x* or *y* is the polarization of the photon field. We will assume *g*_EM_ to have values of 0.05, or 0.10 meV here. As we are treating a many-level system with some transitions in resonance with the photon field and others not, we will not use the rotating wave approximation for the electron–photon interactions in the central system.

The central system is coupled to the leads, functioning as electron and energy reservoirs, by the Hamiltonian

[7]
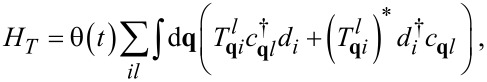


where *d_i_* is an annihilation operator for the single-electron state 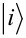 of the central system, *c***_q_***_l_* an annihilation operator for an electron in lead *l* 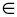 {L,R} in state 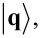 with **q** representing the momentum *q* and the subband index *n_l_* in the semi-infinite quasi-one dimensional lead. The coupling tensor 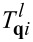 is constructed assuming a nonlocal overlap of the single-electron states at the internal contact boundary of the central system and the respective lead [18–20], extending approximately one effective magnetic length *a_w_* into each subsystem. This approach describing a weak tunneling coupling of the central system and the leads allows for full coupling between the quantum dots and the rest of the central system, like in a scattering approach [21]. Moreover, it conserves parity of states in the transition between the leads and the system. All details and parameters of the coupling scheme have been described in Equations 13 and 14 and the caption of Figure 6 in [20]. The remaining overall coupling constant to the leads is 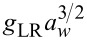 = 0.124 meV, in the weak coupling limit used here.

We will investigate here the physical properties of the open system in the intermediate time range where radiative transitions are active and touch upon the long time evolution [16]. We thus revert to a description based on a Markovian version of a Nakajima–Zwanzig generalized master equation [22–23] that has been derived constructing the kernel of the integro-differential equation up to second order in the lead-system interaction (Equation 7).

We assume a leaky photon cavity described by weakly coupling the single cavity photon mode via the lowest order dipole interaction to a reservoir of photons. For this interaction we assume a rotating wave approximation. Care has to be taken in deriving the corresponding damping terms for the master equation as they have to be transformed from the basis of non-interacting photons to the basis of interacting electrons and photons (the eigenstates of *H*_S_ (Equation 2)) [24–28]. In the Schrödinger picture used here this can be performed by neglecting all creation terms in the transformed annihilation operators and all annihilation terms in the transformed creation operators. This guarantees that the open system will evolve into the correct physical steady state with respect to the photon decay. The Markovian master equation for the reduced density operator ρ_S_ has the form

[8]
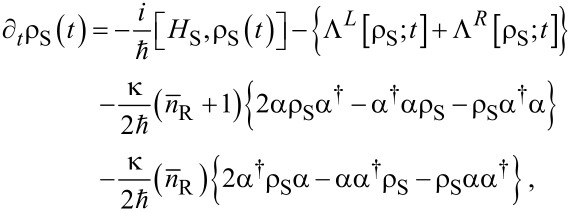


where the creation and annihilation operators α^†^ and α represent the original operators in the non-interacting photon number basis, *a*^†^ and *a*, transformed to the interacting electron photon basis 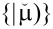 using the rotating wave approximation. We select the photon decay constant as κ = 1.0 × 10^−5^ meV, and 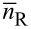 = 0 or 1. The electron dissipation terms, Λ*^L,R^*[ρ_S_;*t*], in the first line of Equation 8 are complicated functionals of the reduced density operator ρ_S_, and are explicitly given in [16] and [29].

We vectorize the Markovian master equation transforming it from the *N*_F_-dimensional many-body Fock space of interacting electrons and photons to a 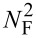-dimensional Liouville space of transitions. The resulting first-order linear system of coupled differential equations is solved analytically [30], and the solution is effectively evaluated at all needed points in time using parallel methods for linear algebra operations in FORTRAN or CUDA [29]. The reduced density operator is used to calculate mean values of relevant physical quantities and the Réniy-2 entropy of the central system [31–33]

[9]
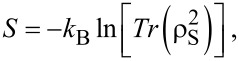


The trace operation in Equation 9 is independent of the basis carried out in, but we use the fully interacting basis 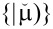 as we do also for the Markovian master equation (Equation 8).

## Results and Discussion

### Many-body states and spectrum

The many-body states of the central system are constructed in a step wise fashion in order to maintain a high accuracy of the numerical results [34]. Initially, a Fock space of non-interacting electrons is constructed from *N*_ses_ = 36 accurate single-electron states (SES) keeping enough one-, two-, and three-electron states in order for the energy of of the highest states for each electron number to surpass the bias window defined by the chemical potential in the leads by a significant amount. The total number of states is 1228 many-electron states (MES) for the selected parameters. This many-body basis is then used to diagonalize the Coulomb interacting (Equation 4) electron system. Second, a basis is constructed as a tensor product of the *N*_mes_ = 120 lowest-in-energy Coulomb interacting electron states and the 16 lowest photon number operator eigenstates. These are subsequently used to diagonalize the closed electron–photon interacting system and create the states 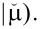 Finally, the lowest *N*_F_ = 120 in energy of these cavity-photon dressed electron states are used for the transport calculation [16]. This step wise construction parallels the step wise construction of Green functions for an interacting electron–photon system.

**Figure 2:**
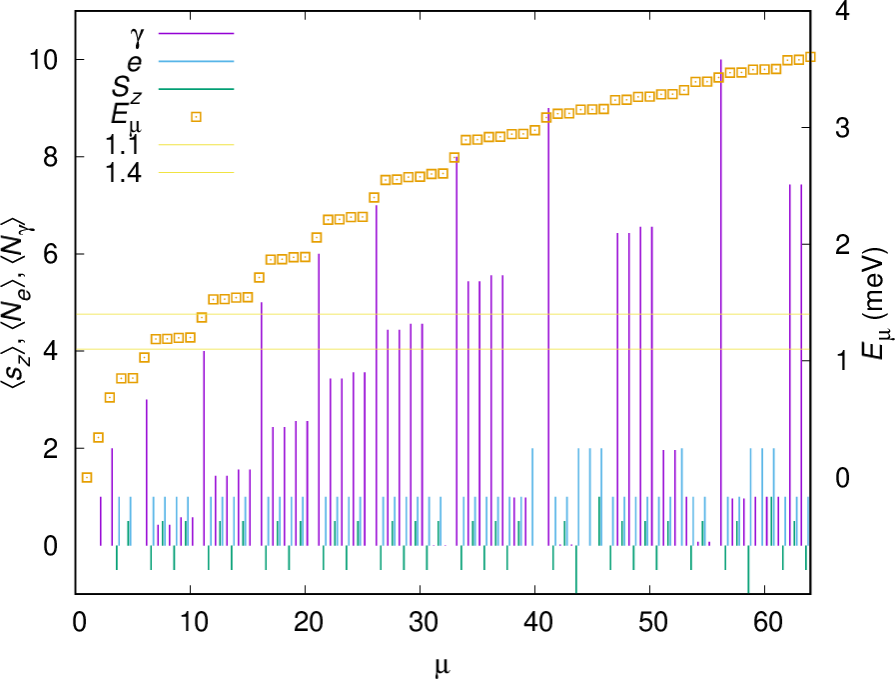
The energy spectrum (gold squares) for the interacting closed central system for plunger gate voltage *V_g_* = 1.6 mV and photon energy 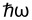 = 0.343 meV as a function of the state number μ. The electron and photon content, and *z*-component of spin is indicated with vertical bars for each many-body state. The chemical potential of the left (μ_L_) and right (μ_R_) leads is shown in relation with the spectrum. *B*_ext_ = 0.1 T, *L_x_* = 180 nm, 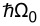 = 2.0 meV, and *g*_EM_ = 0.05 meV. An *x*-polarized cavity photon field is assumed.

The many-body energy spectrum, the electron and the mean photon content, and the *z*-component of the spin of the 64 lowest-in-energy many-body states 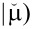 of the closed central system are displayed in Figure 2.

**Figure 3:**
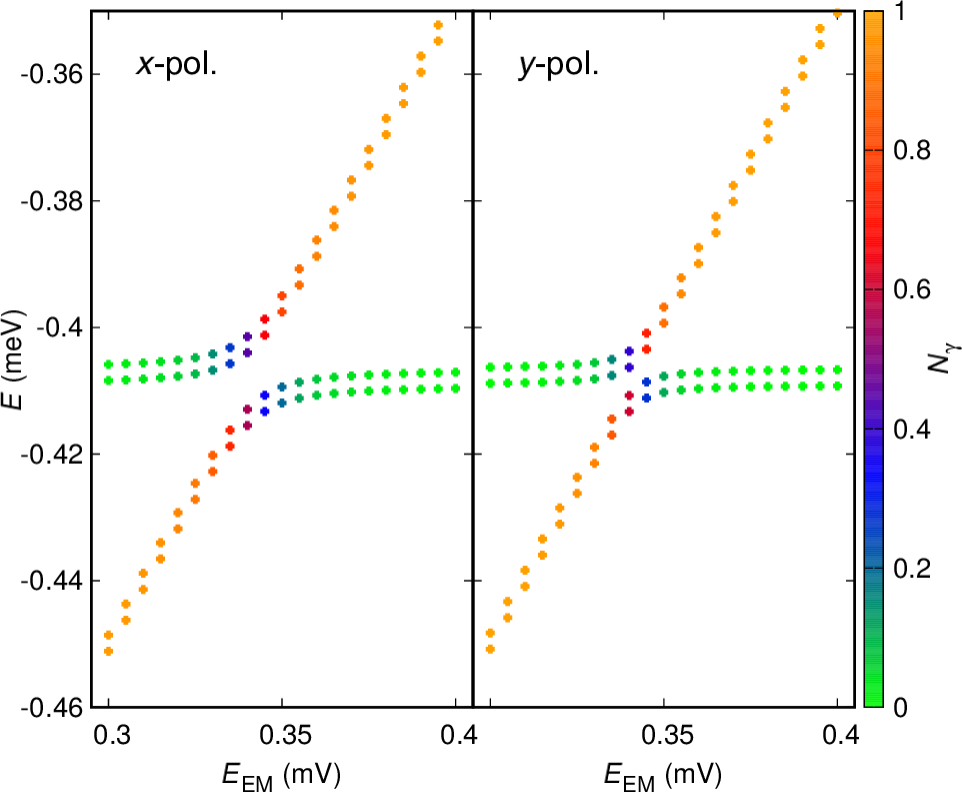
The energy of the two spin components of the lowest in energy one-electron states, mostly localized in the left dot (shallower) and the first photon replica of the corresponding states in the right dot (deeper) as the energy of the cavity photon *E*_EM_ = 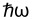 is varied. *V_g_* = 0 mV. For *V_g_* = 1.6 mV the states are labeled with 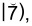 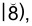 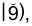 and 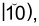 see Figure 2. The mean photon content of the states is indicated by the color of the dots and defined by the color bar on the right side of the figure. (Left) *x*-polarized, and (right) *y*-polarized cavity photon field. *B*_ext_ = 0.1 T, *L_x_* = 180 nm, 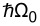 = 2.0 meV, and *g*_EM_ = 0.05 meV.

The photon energy 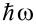 = 0.343 meV coupling the two lowest one-electron states mostly localized in each quantum dot leads to a Rabi resonance showing up in non-integer values for the photon content of some states. The probability density for both spin components of the one-electron ground state are almost entirely localized in the deeper quantum dot, the right dot, but due to the finite separation of the dots, there is a very small probability for the electron to be found in the left dot. The corresponding one-electron wavefunction has positive parity with respect to the dots. These states, 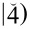 and 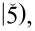 have energies 0.8496 and 0.8521 meV, respectively, and their first photon replicas interact with the two-spin components of the lowest energy one-electron state, mostly localized in the left quantum dot. (The tiny overlap of the charge distribution between the dots causes the corresponding single-electron wavefunction to have negative parity with respect to the dots). The four resulting states 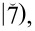 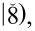 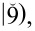 and 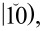 all end up in the bias window defined by the chemical potentials of the left (L) and right (R) leads when the system is opened up for transport. Their energy as functions of the photon energy *E*_EM_ = 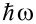 is shown in Figure 3 for an *x*-polarized cavity photon field (left panel) and a *y*-polarized cavity photon field (right panel). The mean photon component of the states and the anticrossings indicates a Rabi splitting that is a bit larger for the *x*-polarized cavity field as the geometry of the system makes the charge densities of the states a bit more polarizable in that direction. As was mentioned earlier, both Rabi splittings are small and not much larger than the Zeeman splitting of the states for *B* = 0.1 T. Due to the weak charge overlap of the states almost localized in each dot, and having opposite parity, both the para- and the diamagnetic electron–photon interactions contribute to the Rabi resonance.

### Transport

**Figure 4:**
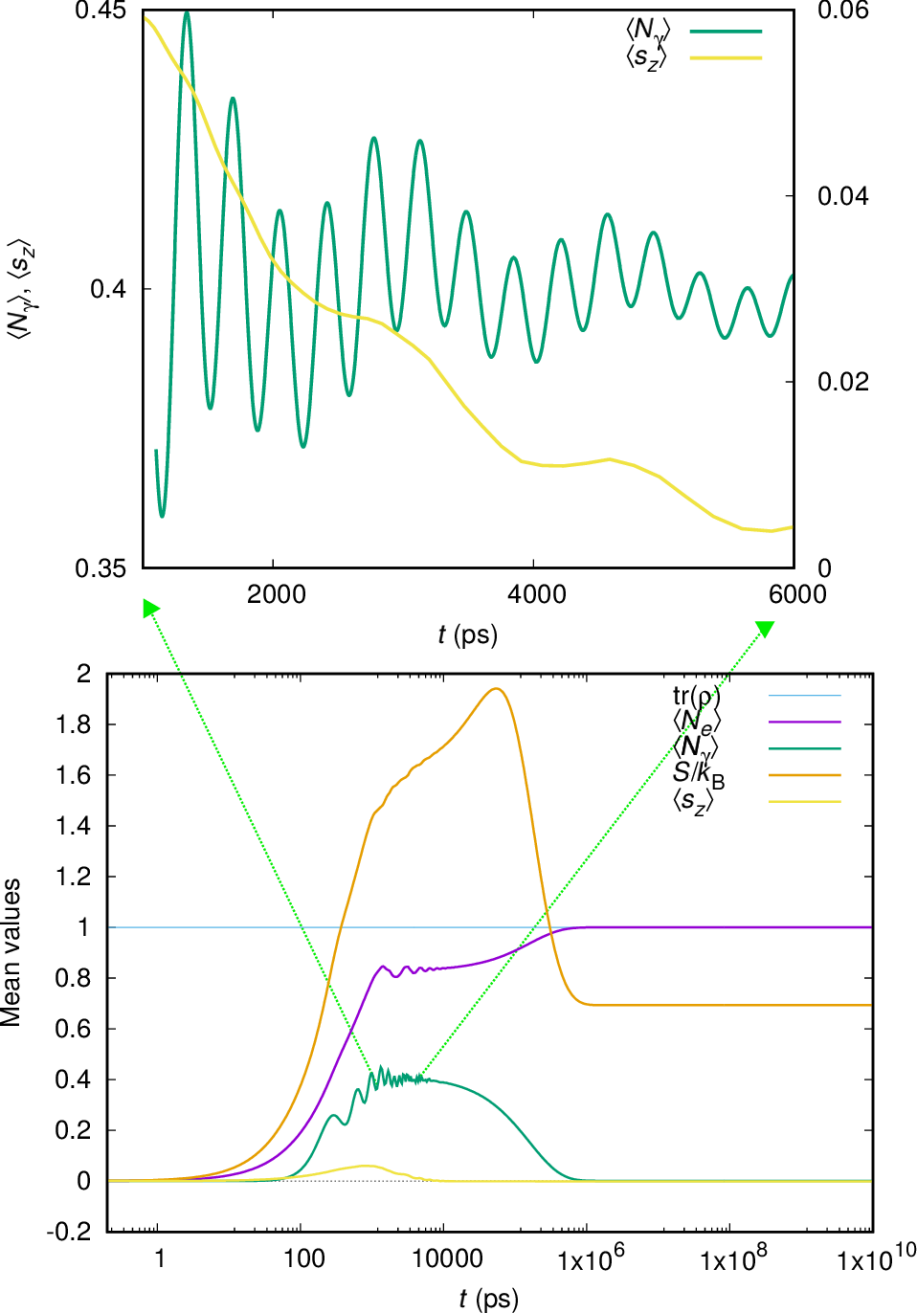
The mean electron 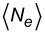 and photon 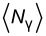 content in the open central system as a function of time. In addition, the mean *z*-component of the total spin, 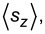 trace of the reduced density matrix, and the entropy of the open central system *S*/*k*_B_ are shown. The upper panel shows the details of 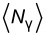 (left *y*-scale) and 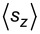 (right *y*-scale) for the intermediate time range. 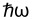 = 0.343 meV, κ = 1.0 × 10^−5^ meV, *B*_ext_ = 0.1 T, *g*_EM_ = 0.05 meV, 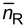 = 0, and *x*-polarized cavity photon.

As stated earlier, Rabi oscillations in the transport current have been predicted in the transient regime [16], and current–current noise spectra in the steady state reveal their signs [15]. Figure 4 displays the mean electron and photon numbers over the whole time scale (lower panel) relevant to the present model parameters for the case of initially empty central system.

In addition, this figure shows the mean value of the *z*-component of the total spin of the electrons, the trace of the reduced density matrix and the Réniy-2 entropy of the central system *S* (Equation 9). Initially, the central system gains electric charge through the states in the bias window. The plunger gate voltage is placed at *V_g_* = 1.6 mV moving the one-electron ground state below the bias window. The steady state is reached when the ground state is fully occupied and the system is Coulomb blocked with no mean current flowing through it. The entropy of the central system starts at zero as it should be for an empty system. This increases in the intermediate time range when many transitions are active in the central system, but does not return to zero in the steady state, which includes both spin components of the one-electron ground state, and is thus not a pure state.

We notice that the mean photon number in the system only assumes a considerable value during the late charging regime from 100 ps to 0.6 μs, when radiative transitions assist in moving charge from the states in the bias window to the ground state of the system [9]. The steady-state photon number vanishes because, on one hand, the cavity is lossy with κ = 1.0 × 10^−5^ meV, and on the other hand, the filling of the single-electron ground state prevents further radiative transitions.

We focus our attention on this intermediate regime, and for a part of it we show the mean photon number and the *z*-component of the total spin in the upper panel of Figure 4. The mean photon number shows oscillations, a faster one that corresponds to the small Rabi splitting energy visible in the left panel of Figure 3, and a slower oscillation that is also present in 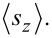 This slower oscillations correlates with the effective Zeeman energy *E*_Z_ = 0.00255 meV at *B*_ext_ = 0.1 T, corresponding to the period *T*_Z_ = 1624 ps. The energy of the cavity photon, 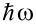 = 0.343 meV corresponds to the time period *T* = 12.1 ps, and is not seen in Figure 4.

**Figure 5:**
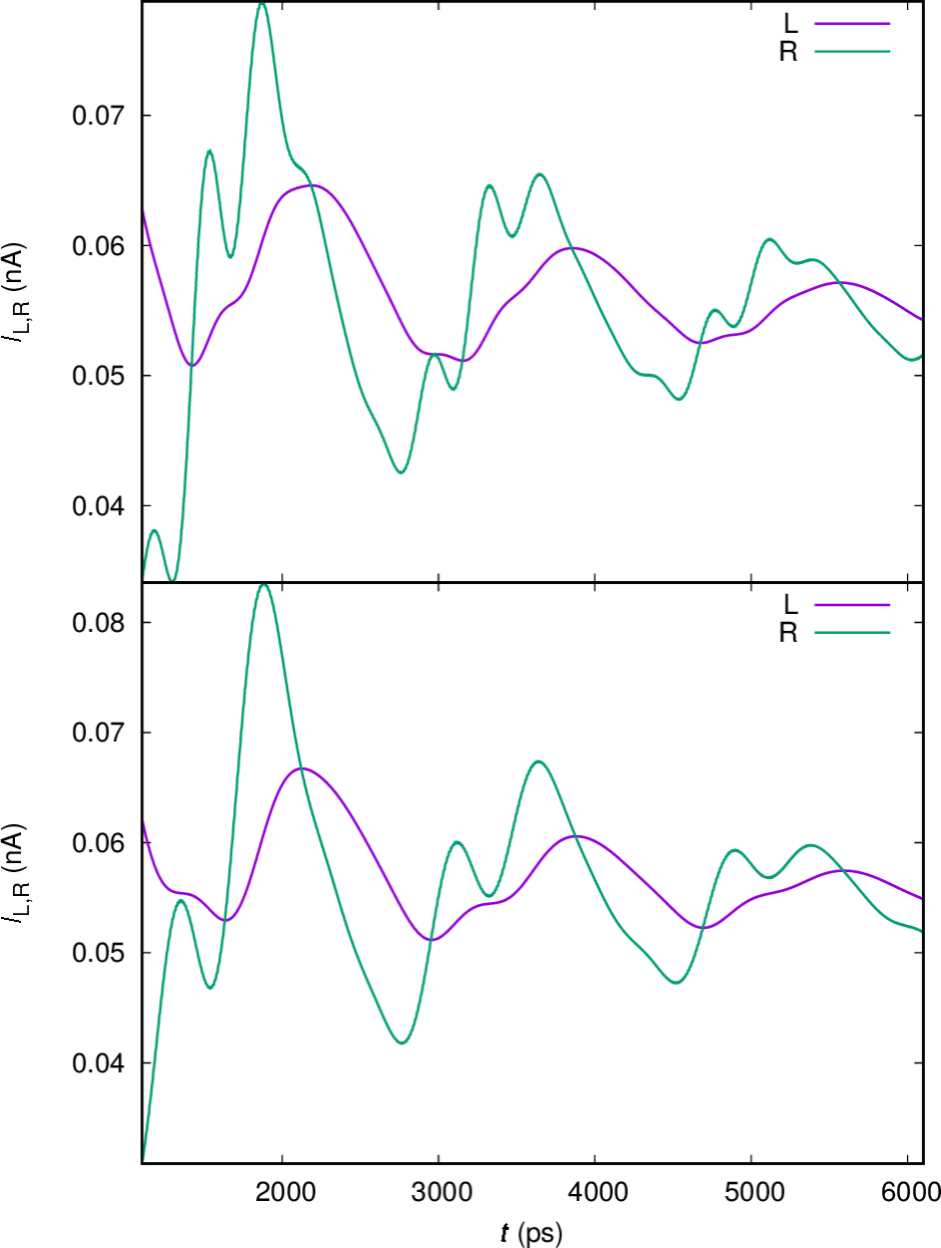
The current from the left lead (L) into the central system and the current from the central system into the right lead (R), for *x*- (upper), and *y*-polarized (lower) cavity photon field. *g*_EM_ = 0.05 meV, 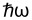 = 0.343 meV, κ = 1.0 × 10^−5^ meV, 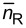 = 0, and *B*_ext_ = 0.1 T.

In order to confirm this identification of oscillations we analyze the left current, *I*_L_, into the central system and the right current, *I*_R_, out of it. The transport current can give us further insight into the dynamics in the system. It is displayed in Figure 5 for the same parameters as were used in Figure 4.

The upper panel displays the current for the *x*-polarized cavity photon field and the *y*-polarized current is shown in the lower panel. The right dot is slightly deeper and wider and its states should have a slightly better coupling to the right lead than the states in the left dot to the left lead. The one-electron ground state is mostly localized in the right dot with its first photon replica in the bias window. Figure 5 shows clear Rabi oscillations in *I*_R_ and much weaker in *I*_L_. The Rabi oscillations are a bit faster for the *x*-polarized photon field than the *y*-polarized in accordance with the Rabi energies readable from the anticrossing levels in Figure 3. Additionally, we notice what seems to be an offset or a phase difference between the left and right current. We address this issue below.

**Figure 6:**
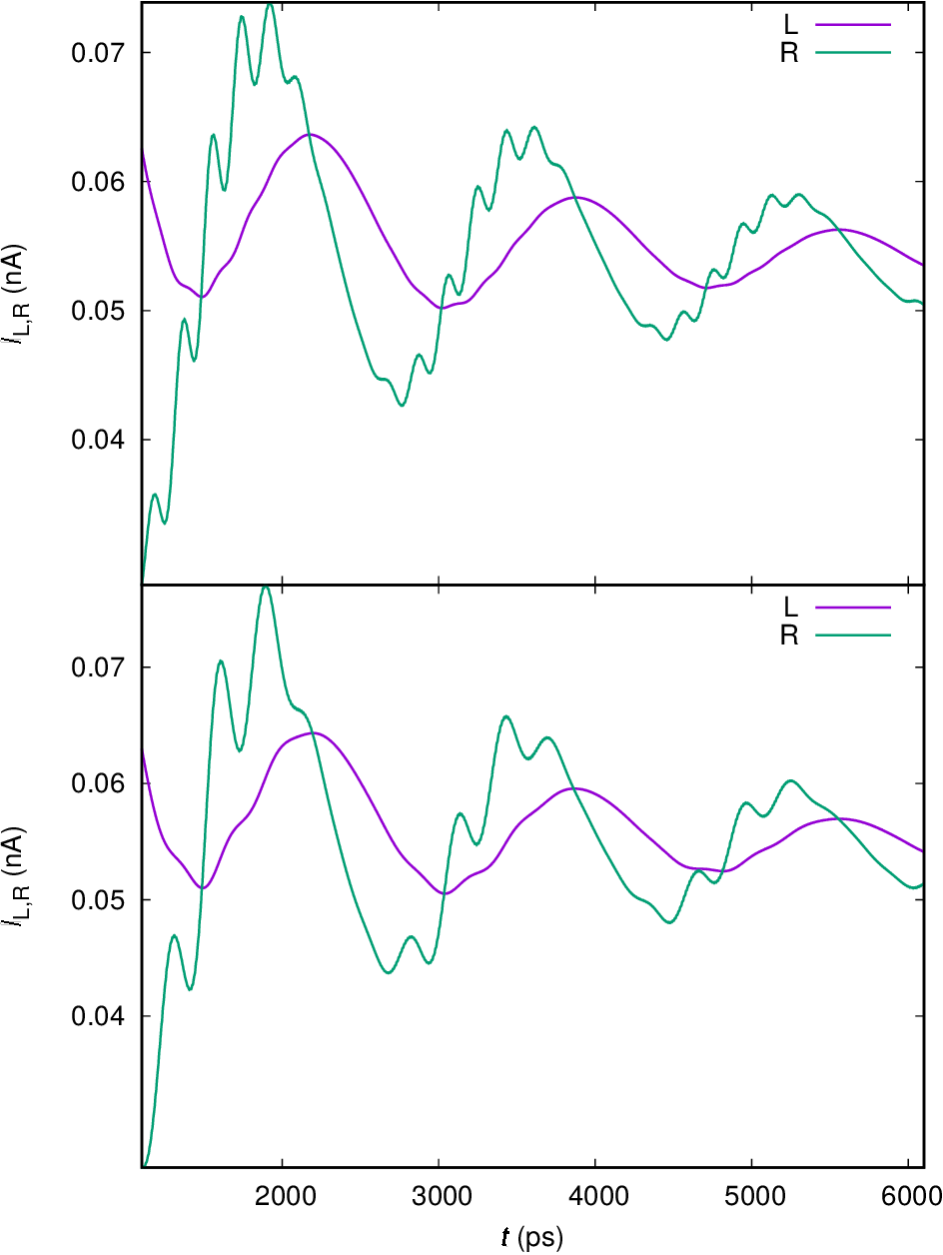
The current from the left lead (L) into the central system and the current from the central system into the right lead (R), for *x*- (upper), and *y*-polarized (lower) cavity photon field. *g*_EM_ = 0.10 meV, 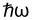 = 0.343 meV, κ = 1.0 × 10^−5^ meV, 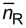 = 0, and *B*_ext_ = 0.1 T.

First, we observe the transport currents for a higher electron–photon coupling in Figure 6, where *g*_EM_ = 0.1 meV, instead of 0.05 meV in Figure 5.

We observe that the Rabi frequency doubles, as expected, for both polarization of the cavity field, but the frequency of the slower oscillations is not changed.

**Figure 7:**
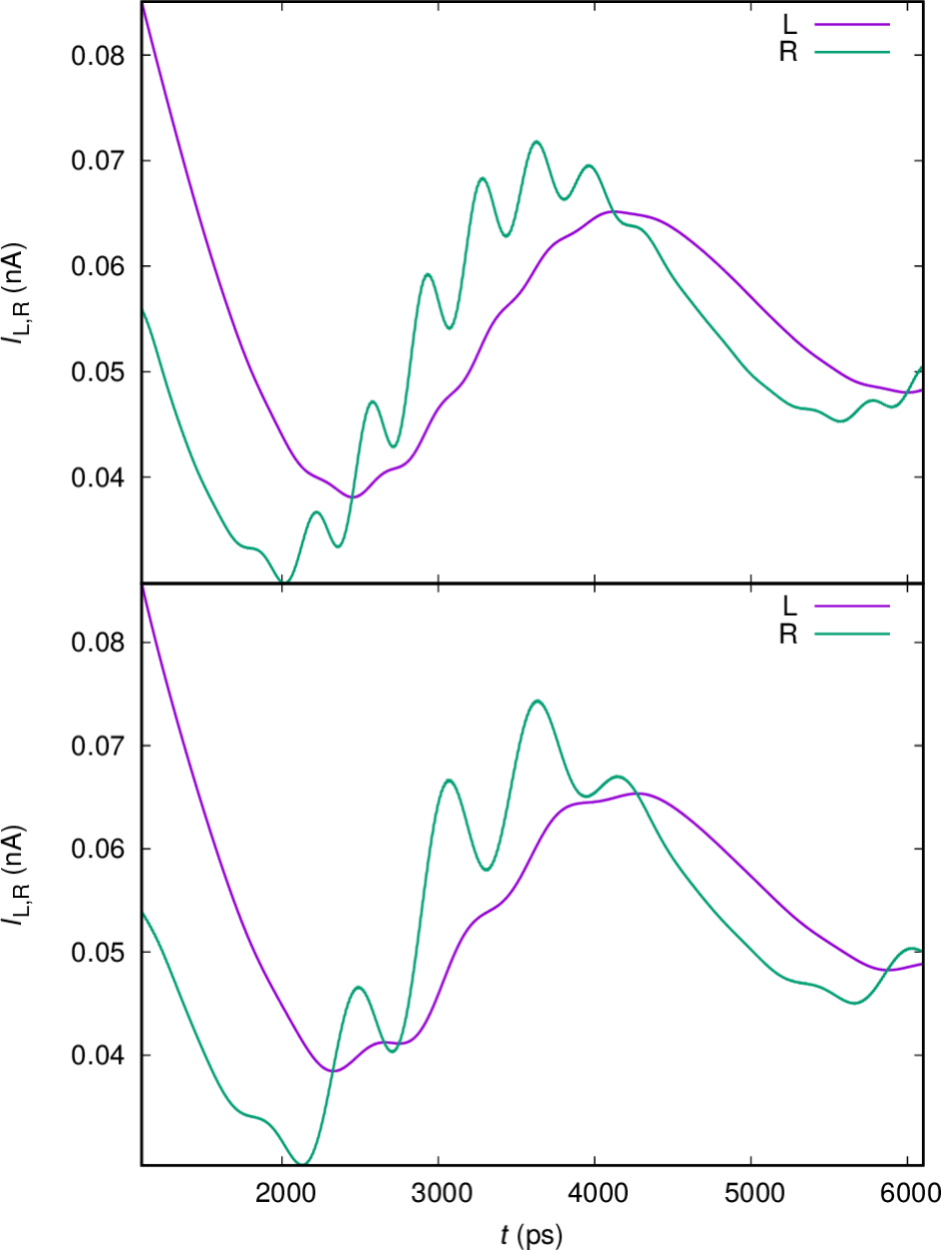
The current from the left lead (L) into the central system and the current from the central system into the right lead (R), for *x*- (upper), and *y*-polarized (lower) cavity photon field. *g*_EM_ = 0.05 meV, 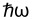 = 0.343 meV, κ = 1.0 × 10^−5^ meV, 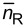 = 0, and *B*_ext_ = 0.05 T.

If the slower oscillations are linked to the Zeeman splitting, then their frequency should change with the small external magnetic field perpendicular to the short quantum wire. In Figure 7 we keep the electron–photon coupling *g*_EM_ = 0.05 meV, but reduce *B*_ext_ from 0.1 T to 0.05 T.

Indeed, the period of the slower oscillation doubles and the faster oscillation remains constant.

**Figure 8:**
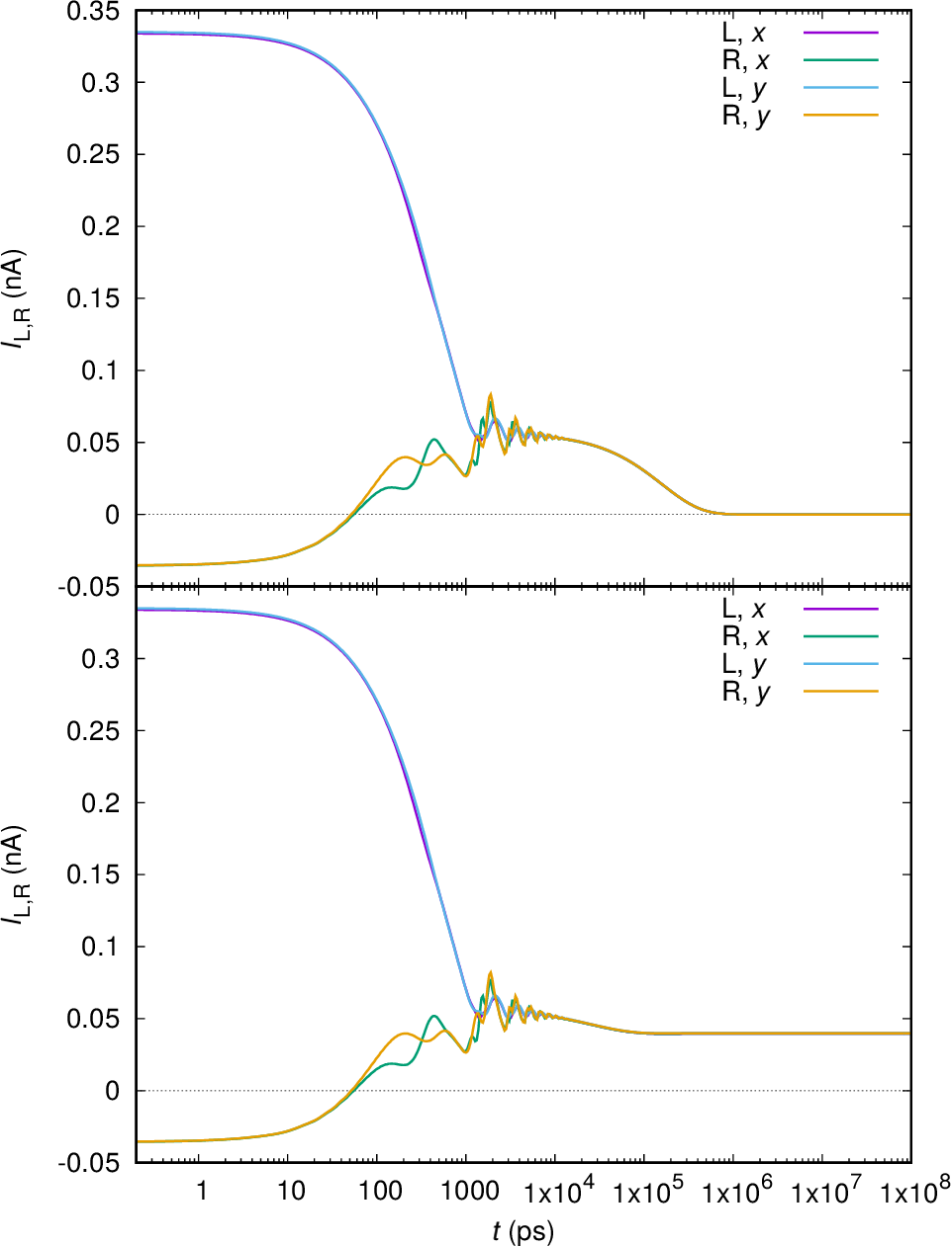
The current from the left lead (L) into the central system and the current from the central system into the right lead (R) for *x*- and *y*-polarized cavity photon field for the whole time range from the transient to the steady state regime. The constant average photon number in the reservoir 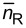 = 0 (upper), and 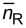 = 1 (lower). *g*_EM_ = 0.05 meV, 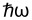 = 0.343 meV, κ = 1.0 × 10^−5^ meV, *B*_ext_ = 0.1 T.

In Figure 8 we show the currents for the whole time scale. In the upper panel we have selected, as above, the photon reservoir to be empty, 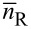 = 0. In this case the system is charged and enters ultimately a Coulomb-blocked steady state with no transport current.

In the lower panel of Figure 8 we assume 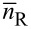 = 1, and in the steady state we have photon-assisted transport. In this case (not shown here) the entropy *S* is not reduced as the system enters the steady state as all photon-active transitions remain active. Clearly seen in Figure 8 is the phase difference between the left and right transport current, even though the logarithmic time scale washes this effect out.

**Figure 9:**
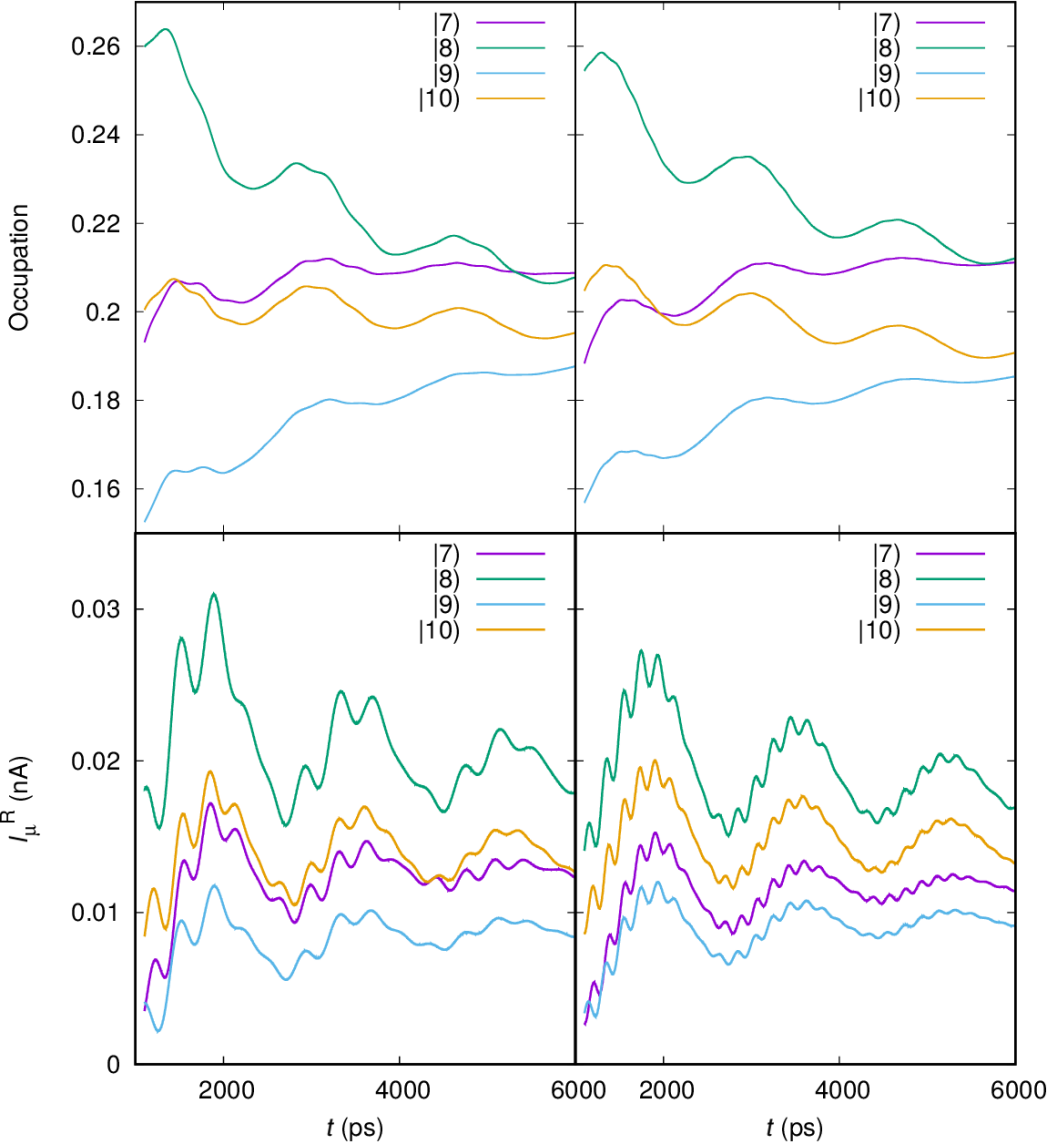
(Upper) The time-dependent partial current through the photon dressed one-electron states 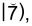 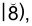 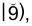 and 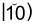 in the bias window for electron–photon coupling *g*_EM_ = 0.05 meV (left), and *g*_EM_ = 0.10 (right). The partial current from the same states from the central system to the left lead for electron–photon coupling *g*_EM_ = 0.05 meV (left), and *g*_EM_ = 0.10 (right). According to Figure 2 states 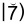 and 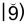 have spin quantum number *s_z_* = −1/2, but states 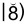 and 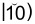 have *s_z_* = +1/2. κ = 1.0 × 10^−5^ meV, *B*_ext_ = 0.1 T, and *x*-polarized cavity photons.

To investigate the reasons for the oscillations with the Zeeman energy we analyze the occupation of the states in the bias window active in the transport in the intermediate time range in the upper panels of Figure 9 for the case of a *x*-polarized cavity field, and two values of the electron–photon coupling, *g*_EM_ = 0.05 meV (left panel) and *g*_EM_ = 0.1 meV (right panel).

The lower two panels of Figure 9 show the partial current through the same states, also for the two different values of *g*_EM_. We will come back to this information below. The partial currents and occupation information are not experimental quantities, but they give us insight into the dynamics in the system. We remember, as is seen in Figure 2 that 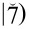 and 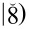 have opposite *z*-components of the spin as do the states 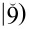 and 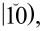 respectively, and we have no spin–orbit interaction in the system. In the upper panels of the figure (Figure 9) we see crossings of the occupation of states with opposite spin. Here, we have to keep in mind that in the intermediate time regime the central system is in nonequilibrium and will evolve to a steady state with a much more intuitive occupation distribution. Moreover, the coupling to the leads of individual many-body states depends on the coupling of their single-particle components, their probability distribution in the contact area of the short quantum wire, and depends on their energy and the density of states of the leads at the corresponding energy. The leads are quasi-1D with a sharply peaked density of states near the subband bottoms. Orbital magnetic effects are included in the leads, but their small Zeeman energy is neglected [18,34]. With all this in mind it is clear that even the coupling of two spin components of the same state to a state in a lead can be different, and the variable occupation of spin levels together with the tiny spin fluctuation seen in Figure 4 during the fastest changes in the system are nonequilibrium fluctuations. Similar can be said for the partial currents shown in the lower panels of Figure 9.

The Rabi resonance for the photon energy 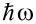 = 0.343 meV entangles the lowest energy one-electron states that are mostly localized in each quantum dot. The time-dependent many-body charge distribution, or electron probability distribution, is thus expected to oscillate between the dots. In Supporting Information File 1 we see the density in a video with 100 frames equally spaced for the time interval *t* = 1102–6000 ps. The video shows oscillations in the charge density between the dots with a combination of the Rabi and the Zeeman frequency. This is in accordance with the left and right transport currents displayed in Figure 5. Moreover, the charge oscillations in the video explain the phase difference between the left and the right transport currents.

**Figure 10:**
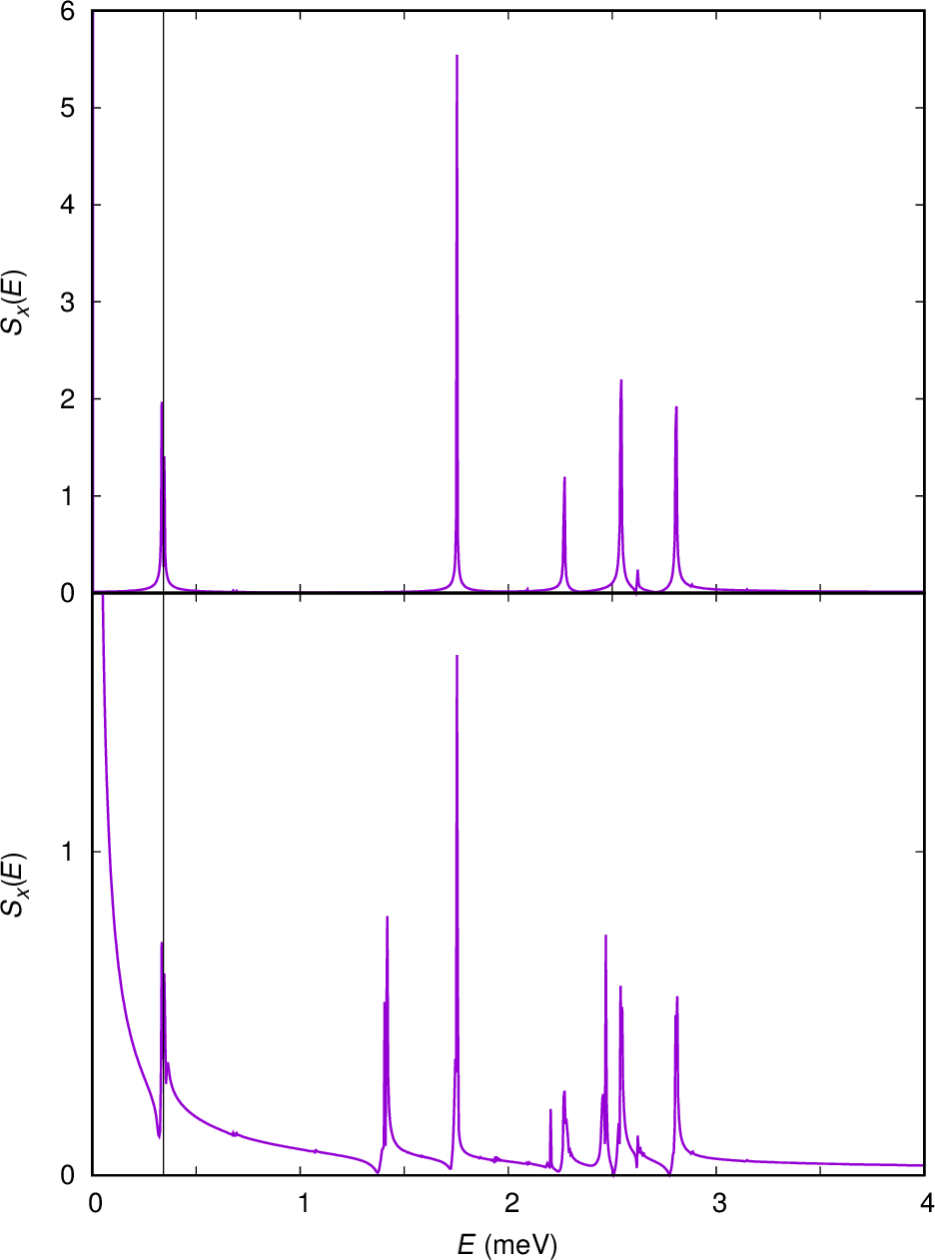
The Fourier power spectrum of the correlation function *S_x_*(τ) = 
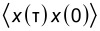
 in the steady state. The cavity photon energy 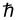 is indicated by a vertical line at 0.343 meV. The constant average photon number in the reservoir 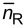 = 0 (upper), and 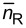 = 1 (lower). *g*_EM_ = 0.05 meV, 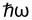 = 0.343 meV, κ = 1.0 × 10^−5^ meV, *B*_ext_ = 0.1 T. The spectra are calculated using 5000 time points in the steady-state regime.

Besides, the oscillations between the dots in the video (Supporting Information File 1) indicate that there might be tiny faster oscillations of the density present within each dot. This is not easy to quantify well within the finite intermediate time range, but can be investigated in the steady state using the correlation function *S_x_*(τ) = 
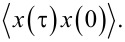
 In Figure 10 we show the Fourier power spectrum of the correlation function *S_x_*(τ) for 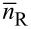 = 0 in the upper panel, and for 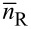 = 1 in the lower one.

The spectra are calculated using the quantum regression theorem [35–36], valid in the Markovian limit for weakly coupled systems [8,37–40]. Both spectra show a peak at the photon frequency 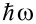 = 0.343 meV and several higher energy peaks that can be assigned to many-body transitions available in the system. The main peak at 1.75 meV is caused by a photon active transition between 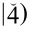 and 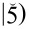 to the states 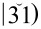 and 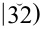 which are the first excitations of both spin components of the one-electron ground state in the right dot. This excitation is thus between states mainly localized in the right dot. The three transitions at 2.2688, 2.54, and 2.808 meV in the upper panel are not photon active transitions to higher states. The additional transitions in the lower panel are mostly additional photon active transitions promoted by the presence of photons in the system.

For 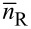 = 0 the system enters a Coulomb-blocked steady state, but for 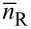 = 1 a photon-assisted transport current flows through it like is seen in Figure 8. Interestingly, “pink” or white noise is seen in *S_x_* when current flows through the system. The same type of noise is seen in the Fourier power spectra of the current–current correlation functions, not shown here. The occurrence of pink or white noise is well known in electronic systems and is here probably caused by a multitude of active transitions for the open multilevel system.

## Conclusion

In summary, we have modeled a nanoscale electron system of two slightly different quantum dots that shows interdot Rabi oscillations between the two lowest energy levels. In the intermediate transient time regime, the Rabi oscillations lead to a phase difference in the currents out of and into the central system. Due to the fast changes in level populations in this regime through radiative and nonradiative transitions, we observe a coexisting spin oscillation even though the electron–photon interactions conserve spin.

The coexistence of the Rabi and the Zeeman oscillations for the intermediate time range is not unique to the present system structure. It has also been observed in a system of two parallel quantum dots embedded in a short quantum wire [13,41]. The main difference between these two cases is the strength of the “interaction” of the quantum dots, or the overlap of the charge densities of the localized states in each quantum dot. For the case of the parallel quantum dots the charge overlap is large to the extent that no eigenstates are localized in either dot, and one might view the system as one highly geometrical, anisotropic quantum dot. In that case, the quantum selection rules make the Rabi resonance between the lowest lying one-electron states to be caused by the paramagnetic electron–photon interaction for a *y*-polarized cavity field, and by the diamagnetic interaction for the *x*-polarization. So, the polarization can be used to change between strong or weak Rabi resonance. Here, that is not the case, as the distance between the dots makes the Rabi resonance rather weak for both photon polarizations.

The master equation used in the model is derived assuming weak coupling of the leads to the central system, to the effect that the kernel of the integro-differential equation is constructed with the system-lead coupling (Equation 7) up to second order. Are we sure this is not producing the oscillations of the occupation of the spin levels? We will likely never be completely sure, but as the time scale for the system needed to attain the steady state shows, we are using a very weak coupling. We have weakened the coupling further, within what is reasonable, as the time scale then becomes even longer, resulting in strain in the numerical accuracy. However, we still see the corresponding spin oscillations. Another indicator is in the paragraph above, i.e., the different effective strengths of the electron–photon interaction, as seen in the different strengths for the Rabi splitting, does not affect the spin-oscillations in time, and neither does a direct change in the strength of the electron–photon interaction, *g*_EM_.

The important message we want to convey from our modeling of time-dependent electron transport through multilevel interacting nanoscale two-dimensional semiconductor systems embedded in 3D photon cavities is that the Rabi oscillations in the central system can be detected in the transport current through them in all of the time regimes characteristic for the corresponding system. Additionally, we observe that the noise spectrum in the steady state depends on whether the system is really open for transport or is in a Coulomb blocking regime.

The challenging terahertz or FIR regime for semiconducting QED circuits offers interesting possibilities for fundamental research into the electron–photon interactions and devices with new potentials.

## Supporting Information

The many-body electron probability density in the central system in the time interval *t* = 1102–6000 ps in 100 frames for an initially empty system. μ*_L_* = 1.4 meV, μ*_R_* = 1.1 meV, 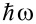 = 0.343 meV, κ = 1.0 × 10^−5^ meV, *B*_ext_ = 0.1 T.

File 1 The time dependent many-body electron probability density in the time interval *t* = 1102–6000 ps.
